# Characterization of Bacterial and Fungal Microbiome in Children with Hirschsprung Disease with and without a History of Enterocolitis: A Multicenter Study

**DOI:** 10.1371/journal.pone.0124172

**Published:** 2015-04-24

**Authors:** Philip K. Frykman, Agneta Nordenskjöld, Akemi Kawaguchi, Thomas T. Hui, Anna L. Granström, Zhi Cheng, Jie Tang, David M. Underhill, Iliyan Iliev, Vince A. Funari, Tomas Wester, HAEC Collaborative Research Group (HCRG)

**Affiliations:** 1 Division of Pediatric Surgery, Departments of Surgery, Cedars-Sinai Medical Center, Los Angeles, California, United States of America; 2 Department of Biomedical Sciences, Cedars-Sinai Medical Center, Los Angeles, California, United States of America; 3 Department of Pediatric Surgery, Astrid Lindgren’s Children’s Hospital, Karolinska University Hospital, Stockholm, Sweden; 4 Department of Women's and Children's Health and Center for Molecular Medicine, Karolinska Institutet, Stockholm, Sweden; 5 Division of Pediatric Surgery, Children’s Hospital Los Angeles, Los Angeles, California, United States of America; 6 Division of Pediatric Surgery, Children’s Hospital Oakland, Oakland, California, United States of America; 7 Genomics Core Laboratory, Medical Genetics Institute, Cedars-Sinai Medical Center, Los Angeles, California, United States of America; 8 Inflammatory Bowel and Immunobiology Research Institute, Cedars-Sinai Medical Center, Los Angeles, California, United States of America; Worcester Polytechnic Institute, UNITED STATES

## Abstract

Development of potentially life-threatening enterocolitis is the most frequent complication in children with Hirschsprung disease (HSCR), even after definitive corrective surgery. Intestinal microbiota likely contribute to the etiology of enterocolitis, so the aim of this study was to compare the fecal bacterial and fungal communities of children who developed Hirschsprung-associated enterocolitis (HAEC) with HSCR patients who had never had enterocolitis. Eighteen Hirschsprung patients who had completed definitive surgery were enrolled: 9 had a history of HAEC and 9 did not. Fecal DNA was isolated and 16S and ITS-1 regions sequenced using Next Generation Sequencing and data analysis for species identification. The HAEC group bacterial composition showed a modest reduction in *Firmicutes* and *Verrucomicrobia* with increased *Bacteroidetes* and *Proteobacteria* compared with the HSCR group. In contrast, the fecal fungi composition of the HAEC group showed marked reduction in diversity with increased *Candida sp*., and reduced *Malassezia and Saccharomyces sp*. compared with the HSCR group. The most striking finding within the HAEC group is that the *Candida* genus segregated into “high burden” patients with 97.8% *C*. *albicans* and 2.2% *C*. *tropicalis* compared with “low burden” patients 26.8% *C*. *albicans* and 73% *C*. *tropicalis*. Interestingly even the low burden HAEC group had altered *Candida* community structure with just two species compared to more diverse *Candida* populations in the HSCR patients. This is the first study to identify *Candida sp*. as potentially playing a role in HAEC either as expanded commensal species as a consequence of enterocolitis (or treatment), or possibly as pathobioants contributing to the pathogenesis of HAEC. These findings suggest a dysbiosis in the gut microbial ecosystem of HAEC patients, such that there may be dominance of fungi and bacteria predisposing patients to development of HAEC.

## Introduction

Congenital aganglionic megacolon, more commonly known as Hirschsprung disease (HSCR), was first described by Harald Hirschsprung in 1887 [1]. The hallmark pathological feature of this condition is the absence of ganglion cells in the distal colon causing a functional bowel obstruction in newborns. Currently, most infants with HSCR are treated with colon pull-through surgery that removes the aganglionic portion of colon and re-establishes bowel continuity in the first weeks of life. Surgical results are satisfactory for most children, however 20–30% of children experience a serious and potentially life-threatening enterocolitis after surgery [2]. Even today, Hirschsprung-associated enterocolitis (HAEC) remains the most frequent complication in children with HSCR resulting in frequent hospitalizations and is the primary cause of mortality in this population [3].

While many etiologies have been proposed for HAEC, the underlying biological mechanisms are poorly understood. A microbial role in the development of HAEC has been suspected since it was first described over 50 years, although at present, no specific organisms have been identified [3]. Both *Clostridium difficile* and rotavirus have been implicated as causative agents of HAEC, however neither were consistently present in patients HAEC [4–6]. With the advancement of molecular microbiological techniques, a PCR based methodology demonstrated that colonization of *Bifidobacteria* and *Lactobacilli* genera were decreased in HSCR patients who developed HAEC, compared with those who did not develop HAEC [7], suggesting that the composition of the bacterial populations may play a role HAEC. Using a genomics approach, Yan *et al*. recently reported characterization of the colonic bacterial microbiome of 4 infants with HSCR, two of which had HAEC [8]. Fecal specimens from multiple regions of the colon were obtained at the time of surgery and showed increased bacterial population diversity in the HAEC patients compared with HSCR patients. These early data suggest the possibility that HSCR children who develop HAEC have a shift from predominant “symbioant” microbes to potentially harmful “pathobioant” referred to as dysbiosis. Dysbioses have been identified as playing an important role in other gastrointestinal conditions such as inflammatory bowel disease [9].

Recently, the intestinal fungal microbiome (or “mycobiome”) has been recognized to coexist with bacteria. Iliev *et al*. showed that by manipulating the commensal colonic fungal population in Dectin-1 knockout mice, the severity of colitis could be either improved or worsened [10]. These findings raise the possibility that the intestinal mycobiome may also influence health and disease in humans.

Herein, we studied 18 HSCR patients from four centers, all of whom had completed definitive surgery to treat HSCR. We compared the colonic bacterial and fungal populations in 9 children with HSCR alone, with 9 children who had at least one episode of HAEC.

## Materials and Methods

### Patients

Inclusion criteria for enrollment were children less than 18 years of age with histopathological diagnosis of Hirschsprung disease who had completed definitive pull-through surgery. Exclusion criteria included presence of intestinal diversion or active HAEC at the time of stool collection, colonic pseudoobstruction and intestinal neuronal dysplasia. This research was approved by the Cedars-Sinai Medical Center IRB (Protocol# 00020809) as a multi-center study, and has been conducted according to the principles expressed in the Declaration of Helsinki. Written informed consent was obtained from a parent by the attending surgeons, research fellows, or research nurses at each site. Children were enrolled by four member institutions of the HAEC Collaborative Research Group (HCRG): Cedars-Sinai Medical Center, Los Angeles, California; Astrid Lindgren Children’s Hospital, Karolinska University Hospital, Stockholm, Sweden; Children’s Hospital Los Angeles, Los Angeles, California; Children’s Hospital of Oakland, Oakland, California. We enrolled 20 children with HSCR, 10 never had a history of enterocolitis and 10 had a history of at least one episode of HAEC based on HAEC scoring system described by Pastor et al [11]. Detailed clinical information was collected using standardized questionnaires that included demographic, medical history, surgical history, radiographic, histopathology, diet, medications (including antibiotics), probiotics and complications. The HCRG data was stored in a secure SQL relational database at the data coordinating center at CSMC.

### Patients excluded from microbiome analysis

Two subjects were excluded from analysis: subject 03–0003 was excluded because he had a diverting ileostomy (that is, the patient had fecal stream diversion after pull-through); and 02–0039 was excluded because he had an active HAEC episode at the time of stool collection. Subject 04–0005 was excluded from fungal microbiome analysis only due to failure to pass quality control after sequencing, although bacterial microbiome analysis was completed.

### Fecal DNA isolation

Within one week of enrollment in the study, stool was collected and frozen at -80°C within 24 hours of collection. All samples were kept frozen and shipped to the coordinating site (CSMC) where all of the samples were prepared for bacterial and fungal DNA[10]. Fecal samples were thawed and resuspended in 50 mM Tris buffer (pH7.5) containing 1 mM EDTA, 0.2% β-mercaptoethanol (Sigma) and 1000 U/ml of lyticase (Sigma). The mix was incubated at 37°C for 30 min and fungal genomic DNA was isolated by using QIAamp DNA Stool Mini Kit (Qiagen) according to the manufacturer's instructions.

### Bacterial and fungal amplicon preparation

Bacterial 16S rRNA gene amplicons spanning variable regions one to four (V1–4) were generated in 20 **μ**L PCR reactions using 20 ng of fecal DNA with 25 cycles using high-fidelity Phusion Polymerase (New England Biolabs, Beverly, MA) at 52.7°C annealing using with degenerate 8F (AGAGTTTGATCMTGGCTCAG) and R357 (CTGCTGCCTYCCGTA) primers. Fungal ITS-1 amplicons were generated in 20 **μ**L PCR reactions using 20 ng of fecal DNA with 35 cycles using Phusion Polymerase at 56.1°C annealing using ITS1F (CTTGGTCATTTAGAGGAAGTAA) and ITS2R (GCTGCGTTCTTCATCGATGC) primers yielded sufficient amplification of ITS targets. All PCR reactions were purified using Agencourt AmPure Magnetic Beads (Beckman), resuspended in 20 μL of nuclease-free water and quantified using a Qubit fluorometer (Invitrogen, Carlsbad, CA).

### Library preparation of bacterial and fungal libraries

Paired-end adapters with unique indexes were ligated to 100 ng of 16S amplicons and used to generate Ion Torrent sequencing libraries using the Ion Xpress Library Kit (Life Technologies, Carlsbad, CA). Illumina paired-end adapters with unique indexes were ligated to 100 ng of ITS-1 amplicons using a modified TruSeq DNA Sample Preparation (Illumina, San Diego, CA) where adapters and PCR primers were diluted 1:10 to accommodate lower input of amplicon mass for both 16S and ITS-1 preparations. Library enrichment was performed with 10 cycles of PCR and purified using Agencourt Ampure Magnetic Beads (Beckman). All libraries were subjected to quality control using qPCR, DNA 1000 Bioanalyzer (Agilent), and Qubit (Life Technologies, Carlsbad, CA) to validate and quantitate library construction then pooled at equimolar concentrations.

### Library sequencing

Pooled libraries were assayed on Agilent Bioanalyzer (Santa Clara, CA) to check final sizing and to check for small fragments as well as KAPA Biosciences qPCR for quantitation. 16S samples were multiplexed and sequenced on the Ion Torrent PGM on a 318 chip with 400bp chemistry. For ITS-1 sequencing final diluted pool was amplified on to a Single End flow cell using clonal bridge amplification on the MiSeq. 250 single-end sequencing-by-synthesis was performed using the MiSeq Illumina sequencer (Illumina, San Diego, CA). The sequence data will be submitted to the NIH Sequencing Read Archive with the submission number: SRP051546.

### Next generation sequencing (NGS) data analysis and species identification

#### Bacterial Species

Ion Torrent reads shorter than 200bp, or not containing the designed 16S primers (>2nt mismatches) were discarded. 300bp sequences of remaining high-quality reads were aligned to the Greengenes reference database (February 2011 release) using BLAST v2.2.22 in QIIME v1.5 wrapper[12] with an identity percentage ≥97% to select the operational taxonomic units (OTUs). Taxonomy for each sequence was assigned using the Ribosomal Database Project (RDP) classifier v2.2.

#### Fungal Species

FASTQ data was de-multiplexed and filtered through a stringent quality control procedure to ensure that only high-quality sequences were analyzed further. To identify fungal species, the filtered reads were aligned with the Findley ITS Database[13] using BLAST v2.2.22 in QIIME v1.5.0 wrapper[12] with an identity percentage ≥97% for OUT picking. Chosen OTUs were compiled into genera or families.

### Diversity Indicies

The original OTU table was randomly subsampled (rarefied) to create a series of subsampled OTU tables. Alpha diversity was calculated in QIIME on each sample using the OTU table and Shannon indices were collated into a single file and the number of species identified for both bacteria and fungi for each sample versus the depth of subsampling was plotted.

### Fungal Quantitative PCR

Fungal reference strain *Candida albicans* (ATCC 90028) was obtained from the American Type Culture Collection (Manassas, VA). Fungi were cultured in aerobic conditions on Sabouraud Dextrose Broth (SDB; EMD Chemicals) for overnight at 37°C. The cultured cells were harvested for DNA preparation using the QIAmp DNA Stool Mini Kit (Qiagen, Inc., USA).

Quantitative PCR was performed on DNA isolated from human stool using SYBR Green Kit (Bio-Rad). Specific primer pairs for *Candida albicans* TTTATCAACTTGTCACACCAGA (Forward) and ATCCCGCCTTACCACTACCG (Reverse); [14]. In a 20ul of qPCR reactive mixture contained 2ul of stool DNA (2~100ng), 10ul of iQ SYBR Green Supermix (2x), 4ul of forward primer (3 pmol/ **μ**L), and 4ul of reverse primer (3 pmol/ **μ**L). The PCR protocol was modified from Iliev et al[10]: Initial denaturation at 94°C for 10 min, followed by 35 cycles of denaturation at 94°C for 30 s, annealing at 55.3°C for 30 s, and elongation at 72°C for 2 min, followed by an elongation step at 72°C for 30 min. *C*. *albicans* in fecal specimens was determined by using a standard curve generated by *Candida albicans* (ATCC 90028) DNA with 10-fold serial dilution from 10^2^ ng to 10^–4^ ng against the threshold cycle C(t), and normalized to the amount of total fecal DNA being used. The qPCR results are reported as *C*. *albicans* cell numbers per ng fecal DNA, in which the *C*. *albicans* cell numbers were calculated from cell counts per nanogram DNA of reference *Candida albicans* (ATCC 90028) where 3x10^-4^ng DNA represents one cell.

### Statistical Analysis

Both OTU sequence numbers and percentages between groups were compared using *Student’s t* test, unpaired, two-tailed, 95% confidence interval. Data are presented as means ± SEM, unless otherwise stated. Statistical analysis was performed using GraphPad Prism 5 software (GraphPad, Inc., San Diego, CA, USA).

## Results and Discussion

### Patient Characteristics

Each group of subjects meeting inclusion criteria consisted of 8 males and 1 female. The median age of all children was 2.7 years (range 5 months to 8 years); the median age of the HSCR group was 2.3 years and the HAEC group was 3.5 years (Tables 1 and 2). Most subjects had aganglionic transition zones in the rectosigmoid colon region; one in the HSCR group had a transverse colon transition zone and one in the HAEC group had an ileal transition zone (total colonic aganglionosis). There were no significant differences in diet (breast milk vs. formula) or probiotic use in the children who developed HAEC compared with those who did not develop HAEC. Three children in the HAEC group received antibiotics within 2 months prior to stool collection: two for treatment of HAEC and one as daily prophylaxis for sickle cell disease, while none of the HSCR group received antibiotics. Not surprisingly, three of the patients in the HAEC group developed HAEC as a complication within the first 30 days after pull-through procedure, while none of the HSCR patients had complications. One patient in the HSCR group had trisomy 21, while two patients in the HAEC cohort had trisomy 21 and one had sickle cell disease.

**Table 1:** Patient Characteristics: HSCR Only.

Subject ID^a^	Sex	Age at stool collection (yr)	Location of TZ^b^		Diet^c^		Abx^e^	Probiotics^f^	Type	Duration (months)	30 day compl^g^	Chr. anomalies^h^
				Breast milk	Duration (months)	Formula						
01–0003	M	3.3	RS	+	3	+	N	Y	LB	9	-	-
01–0006	M	5.3	TV	-		+	N	N	-		-	-
02–0035	M	0.4	RS	+	5	-	N	Y	Lr	4	-	Ts 21
02–0036	M	0.8	RS	+	9	-	N	Y	Lr	6	-	-
02–0040	M	0.2	RS	-		+	N	Y	Lr	Unk	-	-
03–0004	M	8	RS	+	12	+	N	N	-		-	-
04–0003	M	2.3	RS	-		+	N	N	-		-	-
04–0004	M	1.9	RS	+	Unk^d^	+	N	N	-		-	-
04–0007	F	2.3	RS	-		+	N	N	-		-	-
03–0003#	M	3.2	DC	-		+	Y*	N	-		IH	-

^a^Sample ID and site identification: 01—Cedars-Sinai Medical Center, Los Angeles, California; 02- Karolinska Institute, Stockholm, Sweden; 03—Children’s Hospital Los Angeles, Los Angeles, California; 04—Children’s Hospital of Oakland, Oakland, California.

^#^ This subject was *excluded* from analysis because this patient had an ileostomy at the time of stool collection.

^b^Location of TZ: location of transition zone from normal to aganglionic bowel. RS = rectosigmoid colon; DC = descending colon; TV = transverse colon.

^c^Diet: Diet in the first year of life

^d^Unk = unknown

^e^Abx: Antibiotics received within the 2 months prior to stool collection for this analysis.

* = Metronidazole

^f^Probiotics: LB = lactobacillus sp. and bifidobacterium sp. combined; Lr = Lactobacillus reuteri; LG = Lactobacillus GG.

^g^30-day compl: Complications occurring within 30 days of the pull-through operation. IH = internal hernia requiring surgical intervention requiring intestinal resection and ileostomy creation.

^h^Chr. Anomalies: Chromosomal or known genetic mutations. Ts 21 = Trisomy 21

**Table 2:** Patient Characteristics: HAEC.

Subject ID^a^	Sex	Age at stool collection (yr)	Location of TZ^b^		Diet^c^		Abx^e^	Probiotics^f^	Type	Duration (months)	30 day compl^g^	Chr. anomalies^h^
				Breast milk	Duration (months)	Formula						
02–0037	M	1.7	RS	+	6	+	Y*	Y	Lr	6	HAEC	Ts 21
02–0038	M	5	RS	-		+	N	Y	Lr	Unk	-	-
03–0001	F	3.5	RS	-		+	Y**	N	-		-	SC
03–0005	M	2	RS	+	24	-	N	N	-		-	-
03–0006	M	7.6	RS	-		+	Y*	N	-		HAEC	-
03–0007	M	5.1	RS	+	3	+	N	N	-		-	-
03–0008	M	3.1	RS	-		+	N	Y	LG	0.5	-	Ts 21
03–0010	M	3.7	RS	+	1.5	+	N	N	-		HAEC	-
04–0005	M	1.2	Il	+	12	Unk^d^	N	N	-		-	-
02–0039#	M	0.5	RS	-		+	Y*	Y	Lr	6	AS	-

^a^Sample ID and site identification: 01—Cedars-Sinai Medical Center, Los Angeles, California; 02- Karolinska Institute, Stockholm, Sweden; 03—Children’s Hospital Los Angeles, Los Angeles, California; 04—Children’s Hospital of Oakland, Oakland, California.

^#^ This subject was *excluded* from analysis because he had active HAEC at the time of stool collection.

^b^Location of TZ: location of transition zone from normal to aganglionic bowel. RS = rectosigmoid colon; Il = ileum.

^c^Diet: Diet in the first year of life

^d^Unk = unknown

^e^Abx: Antibiotics received within the 2 months prior to stool collection for this analysis.

* = Metronidazole;

** = Penicillin prophylaxis daily for sickle cell disease.

^f^Probiotics: LB = lactobacillus sp. and bifidobacterium sp. combined; Lr = Lactobacillus reuteri; LG = Lactobacillus GG.

^g^30-day compl: Complications occurring within 30 days of the pull-through operation. IH = internal hernia requiring surgical intervention requiring intestinal resection and ileostomy creation. HAEC = Hirschsprung-associated enterocolitis. AS = anastomotic stricture requiring dilatation.

^h^Chr. Anomalies: Chromosomal or known genetic mutations. Ts 21 = Trisomy 21; SC = Sickle cell disease.

### Bacterial microbiome analysis

A mean of 16,304 sequences per sample were analyzed and an estimate of diversity in each group using rarefaction curves suggested greater diversity of bacterial species in the HAEC group compared with the HSCR patients (S1 Fig, S1 Table).

**Fig 1:**
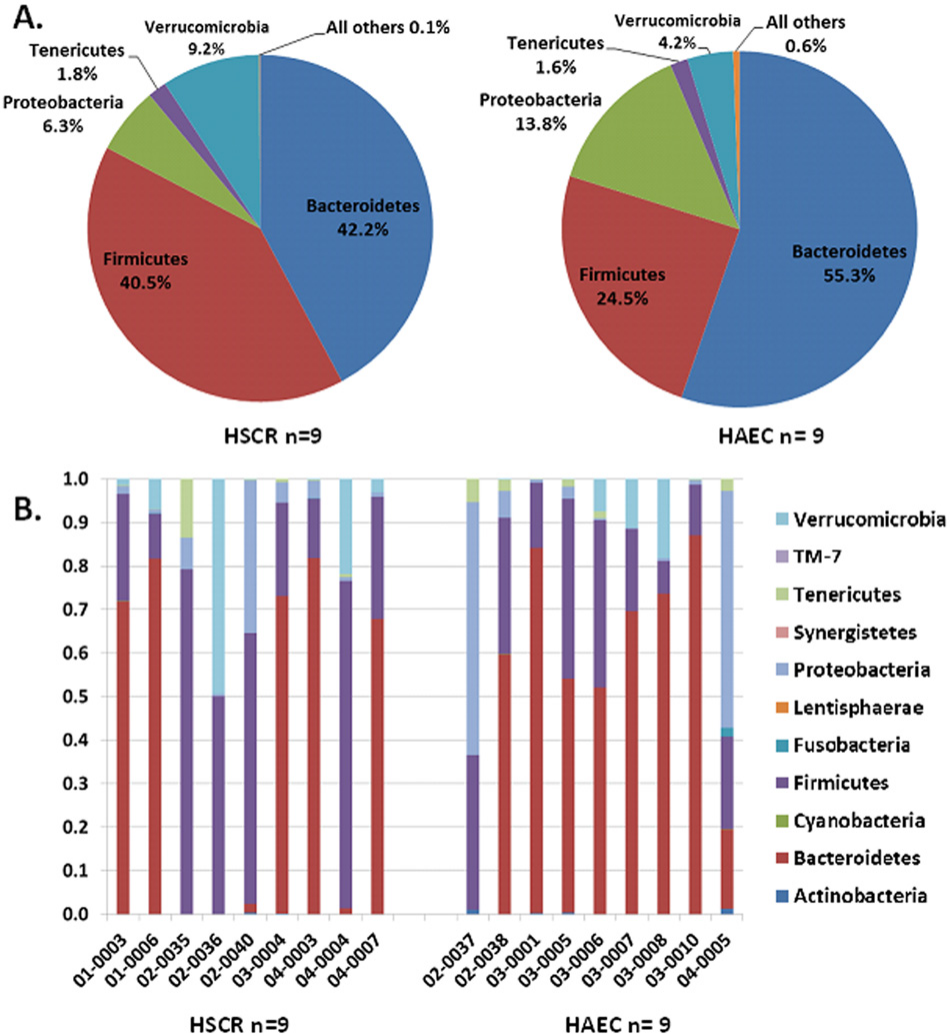
Bacterial phyla in HSCR and HAEC patients. A,16S rRNA gene sequence of fecal bacteria of nine HSCR patients and nine HAEC patients. The pie charts show average relative abundance of five major phyla and six subdominant phyla (summarized as “All others”). B, histograms demonstrating the phyla level bacterial composition of individual subjects with HSCR and HAEC. Individual subject numbers are labeled on the X axis and expressed as relative OTU abundance per each subject. Colors were assigned for each of the 11 detected phyla with the scheme at the right side.

We next analyzed differences in the proportion of bacterial groups at the phylum level. Five phyla (*Firmicutes*, *Bacteroidetes*, *Proteobacteria*, *Verrucomicrobia* and *Tenericutes)* dominated the bacterial microbiota in most samples (Fig 1A). The proportion of *Firmicutes* and *Verrucomicrobia* was lower in HAEC patients than in HSCR. We observed a lower proportion of *Firmicutes* and *Verrucomicrobia*, at 24.5% and 4.2% and a relative increased proportion of *Bacteroidetes* and *Proteobacteria*, at 55.3% and 13.8%, respectively in the HAEC group, when compared with the HSCR group of 40.5% and 9.2% for *Firmicutes* and *Verrucomicrobia*, respectively, and 42.2% and 6.3% for *Bacteroidetes* and *Proteobacteria*, respectively. Statistical comparisons for each phylum were performed and did not reach significance. The taxonomic composition of 11 phyla for each patient was performed and is shown in Fig 1B. There appears to be clustering of composition by study site as noted in 02–0035, 02–0036, 02–0040 and 02–0037 from the Swedish cohort showing a reduction in proportion of *Bacteroidetes*, which is of unclear significance.

We further analyzed the differences in proportion at the genus level (S2 Fig) and similarly found no statistically significant differences between the groups (S2 Table).

### Fungal Microbiome Analysis

At present, nothing is known about what commensal fungi populate the gut of children with HSCR or how they might contribute to HAEC. A mean of 168,805 sequences were generated per patient and an estimate of diversity in each group using rarefaction curves suggested reduced diversity of fungal species in the HAEC compared with the HSCR patients (S1 Fig, S1 Table).

**Fig 2:**
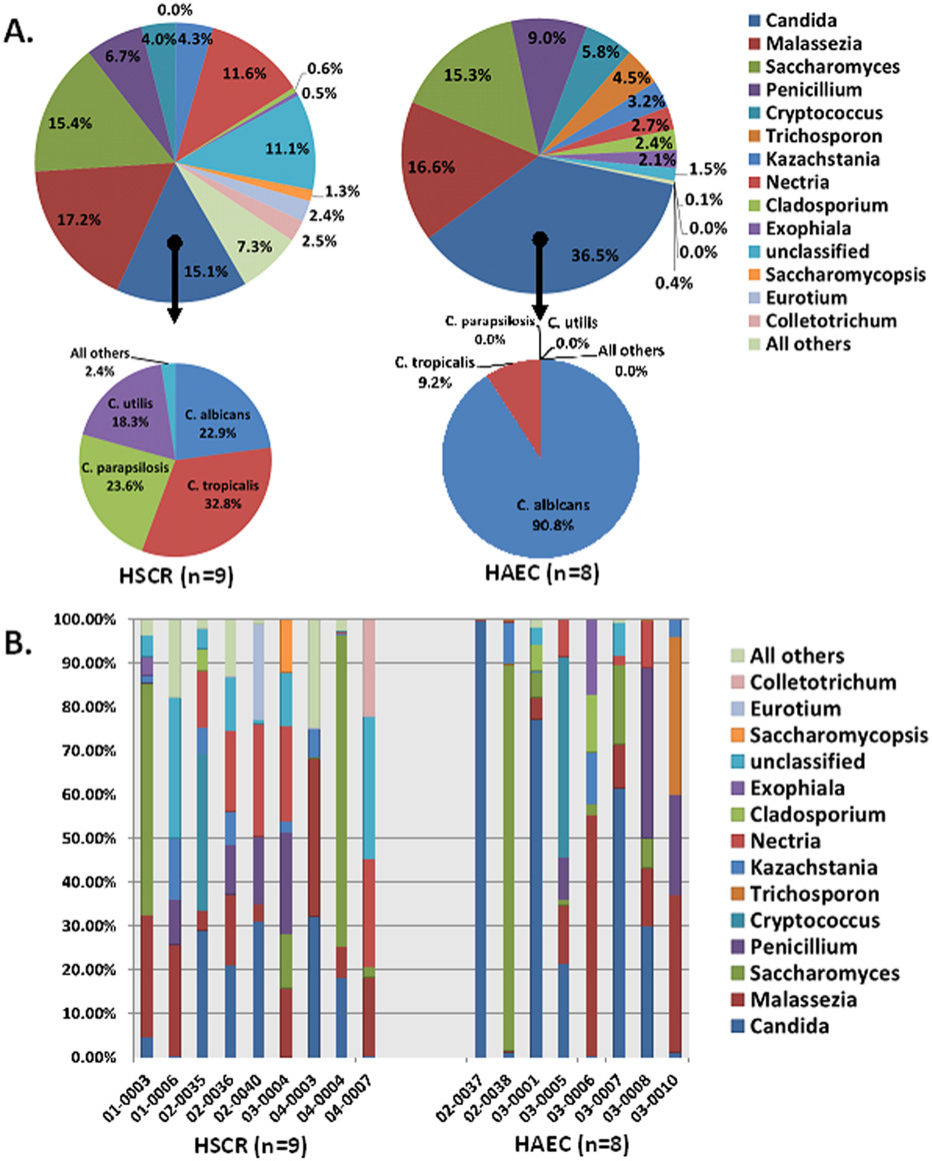
Fungal genera of HAEC patients have increased *Candida sp*. abundance compared with HSCR patients. A, Genera level distribution of fungi in nine HSCR patients and eight HAEC patients expressed as OTU abundance of 18S ITS-1 sequences (upper panel). *Candida* species composition in HSCR and HAEC patients (lower panel). B, Histograms demonstrating the fungal genera composition of individual subjects with HSCR and HAEC. Individual subject numbers are labeled on the X axis and expressed as relative OTUs abundance per each subject. Seventy-four different genera were identified by ITS-1 sequencing. The histogram shows 13 most abundant genera, unclassified genera, and 61 infrequent genera being summarized as “All others”.

**Fig 3:**
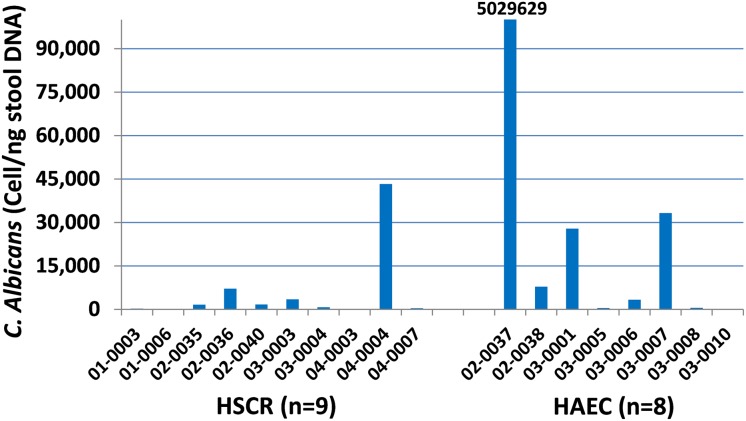
Quantitative PCR of *Candida albicans*. The quantitation of *C*. *albicans* by quantitative PCR on total fecal DNA from HSCR and HAEC patients.

Detailed analysis identified 74 different well-annotated fungal genera, which illustrated the fungal diversity. Note that 89–98% of all fungal sequences identified belonged to 11 fungal genera in the samples analyzed. In HSCR group, we found that 15.1% of the sequences belong to *Candida*, while the HAEC group had a considerably larger portion at 36.5% of sequences (Fig 2A, upper pie chart). The *Candida* species of the HSCR group was split between *C*. *albicans*, 22.9%; *C*. *tropicalis* 32.8%; *C*. *parapsilosis* 23.6%; *C*. *utilis* 18.3% while the HAEC patients had an overwhelming majority of *C*. *albicans* 90.8% and low *C*. *tropicalis* 9.2%, such that 33% of sequences belong to *C*. *albicans* (Fig 2A, lower pie chart). The taxonomic composition of the 13 most abundant fungal genera for each subject was performed and is shown in Fig 2B, and not surprisingly, *Candida* was more abundant in the majority of the HAEC patients, but not all. To confirm the dramatically increased *C*. *albicans* observed in the HAEC group, quantitative PCR was performed for *C*. *albicans* in the same specimens and shown in Fig 3. Three of eight HAEC patients showed especially elevated *C*. *albicans*, while only 1 of 9 HSCR patients showed elevated *C*. *albicans*. Subject 03–0010 had such low quantities of *C*. *albicans* DNA in the sample that no amplification was detected.

**Fig 4:**
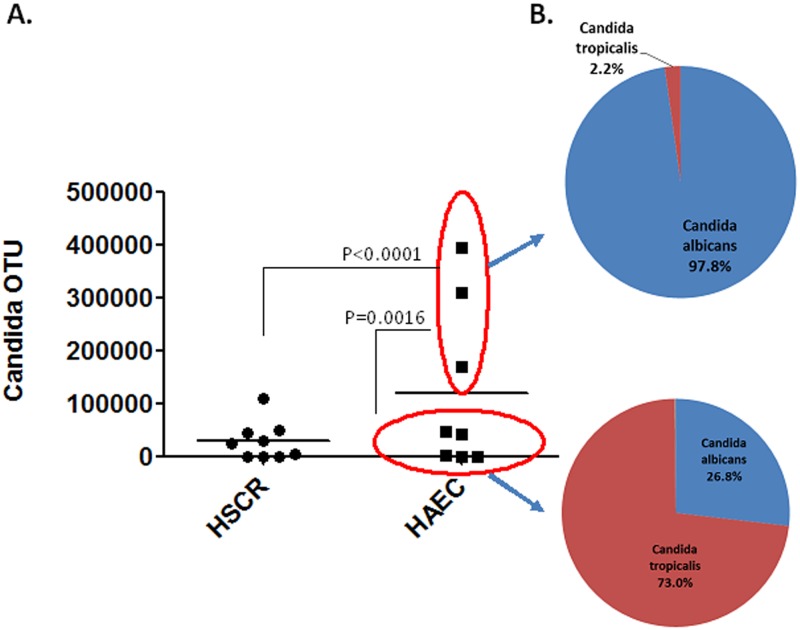
*Candida albicans* and *tropicalis* OTU abundance by phenotype. A, The OTU abundance of *Candida* in feces of HSCR and HAEC patients. Three out of eight HAEC patients showed very elevated Candida OTU’s. B, relative distribution of *C*. *albicans* to *C*. *tropicalis* in the “high burden” patients compared with the “low burden” patients. Comparisons between each group using *t*-test are noted. There was no significant difference between the HSCR and HAEC-*Candida* low burden groups.

When the HSCR and HAEC groups were further analyzed for *Candida* OTU abundance, not surprisingly the identical three HAEC patients showed a high burden of *Candida* compared with the other HAEC and HSCR patients (Fig 4A). There were statistically significant differences in *Candida* OTU abundance between both the HAEC “high burden” (285900 ± 64620) and HSCR (37550 ± 11210) (P<0.0001) and HAEC “low burden” patients (18910 ± 10830) (P = 0.001), respectively, but not between HSCR and HAEC “low burden” patients. In the HAEC group, the species composition of “high burden” patients showed 97.8% was *C*. *albicans* and only 2.2% *C*. *tropicalis* compared with “low burden” patients 26.8% *C*. *albicans* and 73% *C*. *tropicalis* (Fig 4B). Interestingly even the low burden HAEC group did have altered *Candida* community structure with just two species compared to more diverse *Candida* populations in the HSCR patients.

The most striking finding in this study is the high burden of *C*. *albicans* in some HAEC patients, which was not found in the HSCR group. Heretofore, fecal fungi have not been studied in Hirschsprung patients, nor has fungi been implicated in playing a role in HAEC. Importantly, we could not identify a shared clinical feature among high fungal burden HAEC patients, in terms of age, location of transition zone, diet, probiotic use, complications, trisomy 21 status or geographic region. Prior treatment with systemic antibiotics is a potential explanation, as antibiotics are known to result in intestinal *Candida* blooms [15,16]. However, recent antibiotic administration does not fully explain these findings because while 2 high burden patients had received antibiotics, so did 2 low burden patients as well (Table 2).

Further, this is the largest study to date comparing the bacterial microbiome composition of children with HSCR to those who had a history of HAEC that demonstrated modest but potentially important differences. The HAEC group showed reduced abundance of the phyla *Firmicutes* and increase in *Bacteroidetes* and *Proteobacteria*, similar to patients with inflammatory bowel disease (IBD)[17–21], and no statistically significant differences between groups were noted in the bacterial genera. These findings suggest similarities in the gut bacterial milieu of HAEC to IBD, which is intriguing given the recent reports of Hirschsprung disease paients with suspected chronic HAEC have been diagnosed with IBD [22,23]. These findings suggest a dysequilibrium in the gut microbial ecosystem of HAEC patients, such that there may be dominance of bacteria and fungi predisposing patients to development of HAEC.

Our findings also raise the possibility that there may be a subset of children with HAEC in whom *C*. *albicans* may be either a commensal species that is expanded as a consequence of enterocolitis (or treatment), or the intriguing possibility that *C*. *albicans* is a pathobioant that may contribute to the pathogenesis of HAEC. While the mechanism leading to the *C*. *albicans* expansion is unclear, it opens up the possibility of an underlying defect in the gut innate immunity of Hirschsprung patients that may predispose these patients to developing HAEC, as has been demonstrated in severe sub-type of UC patients [10]. Despite the preliminary nature of these findings, one must consider that antifungal therapy may be a novel and rational therapy in selected patients with HAEC.

One limitation of this study is a lack of long-term antibiotic history (prior to 2 months), which may influence the structure of mycobiome. This may confound the interpretion of these results especially in HAEC patients who may have received antibacterials to treat HAEC remotely compared with HSCR patients who have not developed HAEC. Another limitation of this study is the limited sample size of each group.

## Conclusions

In summary, we recognize that the microbiota differences between HSCR and HAEC groups may be caused by treatment; may be caused by HAEC; and may, or may not, actually contribute to HAEC. Nevertheless, we believe that these changes are part of the phenotype of these patients and warrant further study. In the future, an expanded study with more patients is needed to confirm findings, as well as development of animal models to further investigate mechanisms by which the bacterial and fungal communities play a role leading to enterocolitis in the unique milieu of Hirschsprung disease.

## Supporting Information

S1 Fig Rarefaction curves of sequencing data. Rarefaction curves showing the Shannon diversity index change with increasing sequencing depth show that the bacterial (top) and fungal (bottom) sequencing of samples from HSCR patients (left) and HAEC patients (right) reached saturated plateau phase. The plateau in each curve estimates the minimum number of sequences necessary to capture diversity.
(TIF)

S2 Fig Bacterial genera in HSCR and HAEC patients. A,16S rRNA gene sequence of fecal bacteria of nine HSCR patients and nine HAEC patients. The pie charts show average relative abundance of 11 major genera and subdominant genera (summarized as “All others”). B, histograms demonstrating the genera level bacterial composition of individual subjects with HSCR and HAEC. Individual subject numbers are labeled on the X axis and expressed as relative OTU abundance per each subject. Colors were assigned for each of the 11 major genera with the scheme at the right side.
(TIFF)

S1 Table Bacterial and fungal fecal microbiome sequence numbers in HSCR and HAEC patients. The 16S and ITS sequence numbers analyzed, including mean.
(DOCX)

S2 Table Relative abundance of dominant bacterial genera in feces of HSCR and HAEC patients. Relative OTU abundance of 16S rRNA gene sequences of fecal bacteria of nine HSCR patients and nine HAEC patients. Comparisons between HSCR and HAEC groups for each genus was performed using *t-test*.
(DOCX)
