# Supplementary figures and images for: Characterization of Bacterial and Fungal Microbiome in Children with Hirschsprung Disease with and without a History of Enterocolitis: A Multicenter Study
Source: PLoS One. 2015 Apr 24;10(4):e0124172. doi: 10.1371/journal.pone.0124172 (PMC4409062; doi:10.1371/journal.pone.0124172)

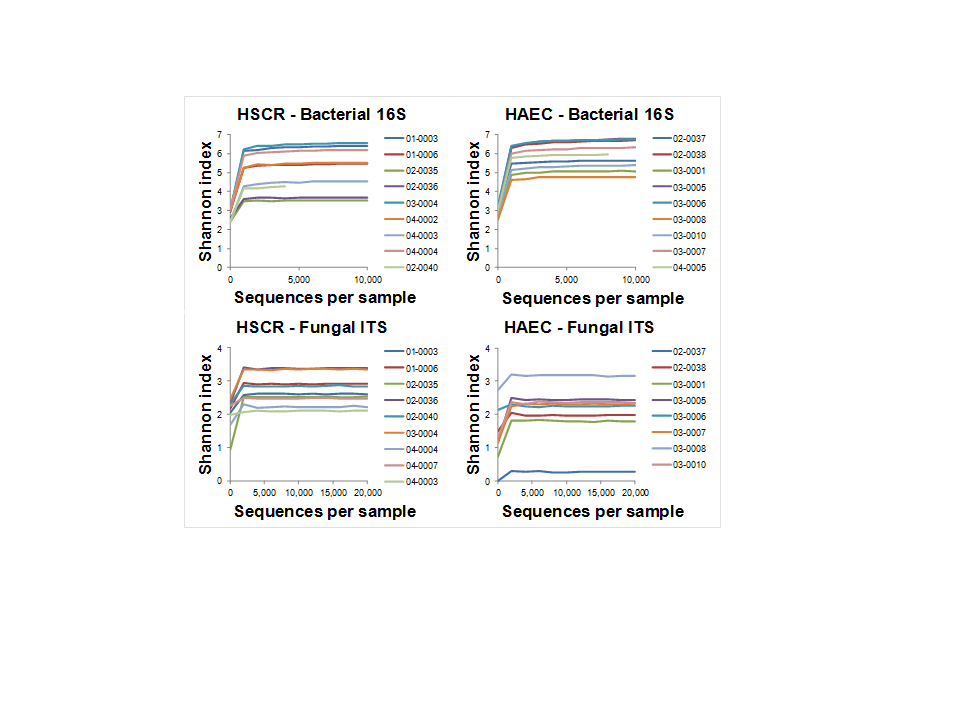

Supplement: S1 Fig — Rarefaction curves showing the Shannon diversity index change with increasing sequencing depth show that the bacterial (top) and fungal (bottom) sequencing of samples from HSCR patients (left) and HAEC patients (right) reached saturated plateau phase. The plateau in each curve estimates the minimum number of sequences necessary to capture diversity. (TIF) [file pone.0124172.s001.tif]

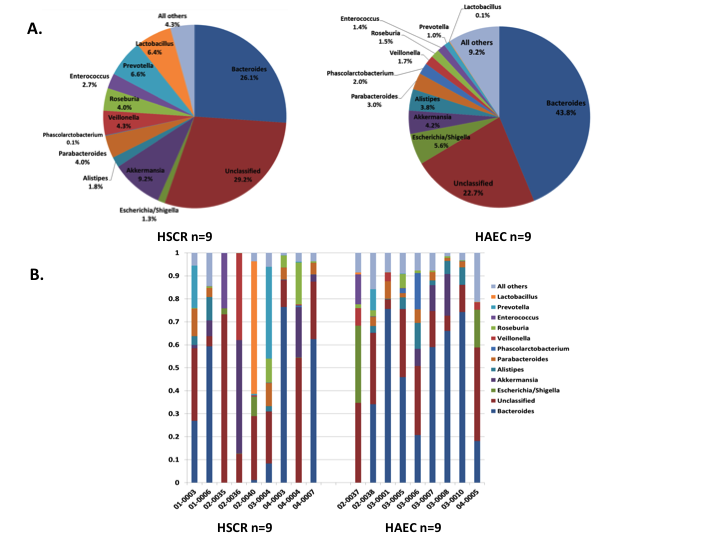

Supplement: S2 Fig — A,16S rRNA gene sequence of fecal bacteria of nine HSCR patients and nine HAEC patients. The pie charts show average relative abundance of 11 major genera and subdominant genera (summarized as “All others”). B, histograms demonstrating the genera level bacterial composition of individual subjects with HSCR and HAEC. Individual subject numbers are labeled on the X axis and expressed as relative OTU abundance per each subject. Colors were assigned for each of the 11 major genera with the scheme at the right side. (TIFF) [file pone.0124172.s002.tiff]
