# Supplementary material for: Characterization of Bacterial and Fungal Microbiome in Children with Hirschsprung Disease with and without a History of Enterocolitis: A Multicenter Study
Source: PLoS One. 2015 Apr 24;10(4):e0124172. doi: 10.1371/journal.pone.0124172 (PMC4409062; doi:10.1371/journal.pone.0124172)
Supplement: S1 Table — The 16S and ITS sequence numbers analyzed, including mean. (DOCX) [file pone.0124172.s003.docx]

**S1 Table. Sequence data of bacterial and fungal fecal microbiome in HSCR and HAEC patients**

|  | **SampleID** | **16S Read Count** | **ITS Read Count** |
| --- | --- | --- | --- |
| HSCR | 01-0003 | 15806 | 102500 |
|  | 01-0006 | 20054 | 96660 |
|  | 02-0035 | 13243 | 139880 |
|  | 02-0036 | 14679 | 130150 |
|  | 02-0040 | 4459 | 293890 |
|  | 03-0004 | 12067 | 225730 |
|  | 04-0003 | 20013 | 55195 |
|  | 04-0004 | 17570 | 227312 |
|  | 04-0007 | 18938 | 115957 |
| HAEC | 02-0037 | 18560 | 339746 |
|  | 02-0038 | 16923 | 20062 |
|  | 03-0001 | 19584 | 333677 |
|  | 03-0005 | 19924 | 177270 |
|  | 03-0006 | 17583 | 87182 |
|  | 03-0007 | 20463 | 221515 |
|  | 03-0008 | 21773 | 111303 |
|  | 03-0010 | 13152 | 191663 |
|  | 04-0005 | 8686 |  |
|  | Minimum | 4459 | 20062 |
|  | Maximum | 21773 | 339746 |
|  | Average | 16304 | 168805 |
