# Supplementary material for: Characterization of Bacterial and Fungal Microbiome in Children with Hirschsprung Disease with and without a History of Enterocolitis: A Multicenter Study
Source: PLoS One. 2015 Apr 24;10(4):e0124172. doi: 10.1371/journal.pone.0124172 (PMC4409062; doi:10.1371/journal.pone.0124172)
Supplement: S2 Table — Relative OTU abundance of 16S rRNA gene sequences of fecal bacteria of nine HSCR patients and nine HAEC patients. Comparisons between HSCR and HAEC groups for each genus was performed using t-test. (DOCX) [file pone.0124172.s004.docx]

**S2 Table**. **Bacterial genera in feces of HSCR and HAEC patients**

|  | HSCR  (Mean ± SD; n=9) | HAEC  (Mean ± SD; n=9) | P value  (Two-tailed t-test) |
| --- | --- | --- | --- |
| *Bacteroides* | 26.1% ± 10.5 % | 43.8% ± 9.0% | 0.2202 |
| *Unclassified* | 29.2% ± 7.3% | 22.7% ± 4.4% | 0.4546 |
| *Escherichia/Shigella* | 1.3% ± 0.9% | 5.6% ± 3.9% | 0.3027 |
| *Akkermansia* | 9.2% ± 5.5% | 4.2% ± 2.2% | 0.4092 |
| *Alistipes* | 1.8% ± 1.1% | 3.8% ± 1.3% | 0.2595 |
| *Parabacteroides* | 4.0% ± 1.5% | 3.0% ± 0.9% | 0.5630 |
| *Phascolarctobacterium* | 0.1% ± 0.09% | 2.0% ± 1.7% | 0.2868 |
| *Veillonella* | 4.3% ± 4.2% | 1.7% ± 0.9% | 0.5456 |
| *Roseburia* | 4.0% ± 2.1% | 1.5% ± 0.6% | 0.2820 |
| *Enterococcus* | 2.7% ± 2.7% | 1.4% ± 1.4% | 0.6881 |
| *Prevotella* | 6.6% ± 4.6% | 1.0% ± 1.0% | 0.2590 |
| *Lactobacillus* | 6.4% ± 6.4 % | 0.1% ± 0.1% | 0.3405 |
| All others | 4.3% ± 1.5% | 9.3% ± 2.0% | 0.0650 |
